# Cases of Poisoning from Chloral

**Published:** 1871-05

**Authors:** 


CASES OF POISONING FROM CHLORAL are reported in a great number of the medical journals, both in Europe and America. In most of the cases it has been used without medical advice.—*Pacific Medical Journal.*

